# Effectiveness of the “Cancer Home-Life Intervention” on everyday activities and quality of life in people with advanced cancer living at home: a randomised controlled trial and an economic evaluation

**DOI:** 10.1186/s12904-016-0084-9

**Published:** 2016-01-22

**Authors:** Å. Brandt, M. S. Pilegaard, L. G. Oestergaard, L. Lindahl-Jacobsen, J. Sørensen, A. T. Johnsen, K. la Cour

**Affiliations:** The National Board of Social Services, 5000 Odense C, Denmark; The Research Initiative of Activity Studies and Occupational Therapy, Research Unit of General Practice, Department of Public Health, University of Southern Denmark, 5000 Odense C, Denmark; OPEN Odense Patient data Explorative Network, Odense University Hospital and University of Southern Denmark, 5000 Odense C, Denmark; Department of Physiotherapy and Occupational Therapy, Aarhus University Hospital, 8000 Aarhus C, Denmark; Institute of Public Health, Aarhus University, 8000 Aarhus C, Denmark; Centre of Health Economics Research (COHERE), Department of Public Health, University of Southern Denmark, 5000 Odense C, Denmark; Department of Psychology, University of Southern Denmark, 5230 Odense M, Denmark; Department of Palliative Medicine, Bispebjerg Hospital, 2400 Copenhagen, NV Denmark

**Keywords:** Palliative care, Supportive care, Health-related quality of life, Activities of daily living, Home based intervention, Occupational therapy, Economic evaluation, Randomized controlled trial

## Abstract

**Background:**

During the past decade an increasing number of people live with advanced cancer mainly due to improved medical treatment. Research has shown that many people with advanced cancer have problems with everyday activities, which have negative impact on their quality of life, and that they spend a considerable part of their time at home. Still, research on interventions to support the performance of and participation in everyday activities is only scarcely available. Therefore, the occupational therapy-based “Cancer Home-Life Intervention” consisting of tailored adaptive interventions applied in the participant’s home environment was developed.

The objective of this study is to examine the effectiveness and cost-effectiveness of the Cancer Home-Life Intervention compared to usual care on the performance of and participation in everyday activities and quality of life in people with advanced cancer living at home.

**Methods:**

The study is a randomised, controlled trial (RCT) including an economic evaluation. The required sample size of 272 adults living at home will be recruited from outpatient clinics at two Danish hospitals. They should be diagnosed with cancer; evaluated incurable by the responsible oncologist; and with a functional level 1–2 on the WHO performance scale. The primary outcome is the quality of performance of activities of daily living. Secondary outcomes are problems with prioritised everyday activities; autonomy and participation; and health-related quality of life. Participants are randomly assigned to: a) The Cancer Home-Life Intervention in addition to usual care, and b) Usual care alone.

**Discussion:**

The trial will show whether the Cancer Home-Life Intervention provides better support for people with advanced cancer living at home in performing and participating in everyday activities, and whether it contributes to their health-related quality of life. The economic evaluation alongside the RCT will show if the Cancer Home-Life Intervention is cost-effective. The trial will also show the acceptability of the intervention to the target group, and whether subgroups of participants will benefit more than others.

**Trial registration:**

ClinicalTrials.gov Identifier NCT02356627. Registered 02/02/2015.

## Background

The number of people living with advanced cancer is increasing [1], and it is estimated that the majority of these people are in need of palliative care [2, 3]. According to the World Health Organization (WHO), some of the goals of palliative care are to alleviate adverse consequences of the disease, to improve the quality of life, and to help patients with life-threatening illness to live as actively as possible [4]. Advanced cancer, defined as cancer diagnosed as incurable by the responsible oncologist, can cause functional limitations [5, 6], which may result in decreased ability and energy to perform everyday activities, such as self-care, household, leisure and work [7–9]. A recent study found that 48 % of patients with advanced cancer (*N* = 977) had problems with everyday activities, and that 29 % had unmet needs regarding these [2]. In another study, more than 43 % of women with metastatic breast cancer (*N* = 163) had difficulties with activities of daily living (ADL) and 74 % with instrumental activities of daily living (IADL) [10]. Further, in a study of people with advanced cancer, 10-30 % reported that they had needs in performing everyday activities, i.e. activities that, in addition to ADL and IADL, include leisure and work [11]. Consequently, many people with advanced cancer are not able to perform needed and desired everyday activities, which may lead to reduced quality of life for the individual [12, 13].

Despite the knowledge about the problems and needs of people with advanced cancer related to everyday activities, palliative care rarely encompasses interventions that focus on enabling everyday activities [14–16], and little is known about how the everyday activities are specifically affected and what kind of support is needed. Prior to the present study, a cross-sectional study was conducted to delineate the everyday activity problems, particularly in regard to ADL performance, activities prioritised to be solved, and intervention needs [17–19]. In all, 164 participants with an advanced cancer, a mean age of 67 years, and a WHO Performance Status of 1–3 were included. The findings showed that many participants had functional limitations such as fatigue and pain, and that their ADL performance was characterised by increased effort and reduced efficiency, safety and independency. About half had ADL motor ability below age expectations. The ADLs that caused most problems were physically demanding activities such as cleaning, laundering, and cooking, while fewer problems with self-care were found [18]. The study showed that the everyday activities which the participants had problems with and prioritised being solved mostly concerned leisure, social and domestic activities, along with a wish for improved mobility, autonomy and participation [18].

A Danish randomised controlled trial (RCT) demonstrated that an activity-focused hospital-based intervention was feasible for people with advanced cancer [20]. The study did not identify superior effect of the intervention partly due to a small sample size [21]. A systematic review has indicated that home-based interventions can improve health-related quality of life and satisfaction with palliative care, reduce depressive symptoms, societal costs, and the number of hospital admissions [22]. In addition, a study has shown that people with advanced cancer spend a considerable part of their time at home [23]. Hence a home-based intervention may be more appropriate than a hospital-based intervention in order to enable everyday activities for people with advanced cancer.

We only identified one home-based intervention study aimed at supporting everyday activities for people with cancer. Hegel et al. performed a pilot RCT of a telephone-delivered problem-solving occupational therapy intervention lasting less than a mean time of 106 min. They found that participation restrictions in everyday activities among rural breast cancer patients undergoing chemotherapy seemed to be decreased due to the intervention [24]. When searching for interventions for groups with activity problems similar to those of people with advanced cancer, such as people with chronic diseases and older people with functional limitations, we only identified few studies. The interventions mainly consisted of adaptation of everyday activities, energy conservation, provision of assistive or mainstream technologies, and home modifications, resulting in improved functioning in everyday activities and quality of life [25–29].

Based on the cross-sectional study, existing studies and available guidelines, a literature review, and consultation with representatives of the target group, the “Cancer Home-Life Intervention” was developed and pilot-tested. The intervention applies adaptive strategies, defined as individual plans to overcome particular challenges or to meet the needs of the study participants [30, 31], and aims to compensate for functional limitations, enhance participation in everyday activities, and support resource/energy preserving activity patterns of people with advanced cancer. The intervention has undergone a small feasibility trial (*N* = 4) showing that the intervention was acceptable for the study participants and possible to implement. Since the Cancer Home-Life Intervention is newly developed an evaluation of its effectiveness and cost-effectiveness is required prior to wider implementation. This study protocol outlines how we intend to conduct such evaluation.

## Study objective

The overall objective of this study is to examine the effectiveness and cost-effectiveness of the Cancer Home-Life Intervention compared to usual care on the performance of and participation in everyday activities and health-related quality of life in people with advanced cancer living at home.

### Specific aims

- To examine the effectiveness of the Cancer Home-Life Intervention in terms of quality of ADL performance as a primary aim and in terms of problems with everyday activities prioritised to be solved; autonomy and participation in the dwelling; and health-related quality of life as secondary aims.
- To investigate whether the Cancer Home-Life Intervention is especially effective in some subgroups of people with advanced cancer defined by age, gender, primary cancer diagnosis, and the WHO Performance score.
- To explore how people with advanced cancer experience the usefulness of the intervention, and how activity patterns change over time in the two groups.
- To investigate the cost-effectiveness of the Cancer Home-Life Intervention.

#### Hypotheses

- The quality of ADL performance as demonstrated by motor and process ability will be better in participants undergoing the Cancer Home-Life Intervention compared to participants who receive usual care.
- Participants undergoing the Cancer Home-Life Intervention will report less difficulty with prioritised everyday activities, better autonomy and participation, and higher levels of health-related quality of life compared to participants who receive usual care.
- The Cancer Home-Life intervention provides more Quality Adjusted Life Years (QALYs) at a higher incremental cost.
- The Cancer Home-Life intervention is cost-effective in a health sector perspective.

## Methods

### Trial design

The study is designed as a RCT using a combination of quantitative and qualitative methods. A health economic evaluation will be performed alongside the RCT.

### Participants

Study participants will be enrolled consecutively from Aarhus University Hospital (OUH) and Odense University Hospital (AUH) in Denmark. Participants who fulfil the following inclusion criteria will be enrolled in the study:

*Inclusion criteria:*

- ≥18 years old
- Diagnosed with cancer
- Evaluated incurable by responsible oncologist in respective out-patient clinic
- Functional level 1–2 on the WHO performance scale as assessed by hospital nurses or the project occupational therapist (P-OT) [32]
- Live within a radius of maximum 60 km from AUH or on the island of Funen
- Live in a private home or in sheltered living
- Know sufficient Danish to complete questionnaires and participate in interviews

*Exclusion criteria:*

- Cognitive impairment preventing the participant to complete the structured interview as assessed by a P-OT during the interview prior to enrolment
- Live in a nursing home or a hospice
- Considered incapable of complying with the trial by a P-OT

### Enrolment procedure

At each participating hospital, nurses, secretaries, or the Palliative Team will screen all potential participants for inclusion in the study during 24 months. When eligible participants are identified, the contact information is given to a P-OT responsible for enrolment of study participants. The P-OT will contact all potential participants and provide detailed verbal and written information about the study. Prior to inclusion, the study participants are to give written permission.

## Intervention and control

### Intervention

The Cancer Home-Life Intervention is described in a detailed intervention manual (unpublished, can be retrieved from the authors) and is provided by trained Intervention Occupational Therapists (I-OT). The intervention program is occupational therapy-based and encompasses individually tailored combinations of the following elements: 1) prioritisation of resources, energy, and everyday activities; 2) adaptation of activities; 3) adaptation of posture and seating positioning; 4) provision of assistive devices; 5) modification of the physical home environment. Intervention elements are selected in co-operation between the participant and the I-OT by means of an interview with the participant based on the individual’s problems and needs. The intervention is provided through instruction in and training of the selected strategies, such as how to conduct activities in energy conserving and strain minimizing ways, and guidance and training in safe and efficient use of assistive devices. It is provided in the participant’s home within a week after baseline data collection and completed within three weeks after the initial home visit. The intervention will encompass 1–3 home visits followed by 1–3 telephone calls. When needed, the participant can also contact the I-OT.

In addition to the Cancer Home-Life Intervention, the participants in the intervention group will receive usual care as offered by the hospital and municipality. Usual care aimed at enhancing everyday activities of people with advanced cancer sometimes consists of provision of assistive devices and home modifications, but not necessarily provided systematically. All participants will be allowed to use available medical services such as rehabilitation and palliative care.

Four I-OTs, two at each hospital, will provide the intervention after having attended a one-day training course in the application of the Cancer Home-Life Intervention. Regular meetings will be held with the I-OTs in order to ensure that the intervention is applied according to the manual and as similar as possible across hospitals. The I-OTs will document adherence to the intervention manual by registering which components of the Cancer Home-Life Intervention they have provided to each participant. The study participants will register in a structured questionnaire which everyday activity enabling interventions they have been offered and report whether they have used them.

### Control

Participants in the control group will receive usual care as offered by the hospital and municipality, described above.

## Instrumentation

### Primary outcome

Quality of ADL performance is measured by *The Assessment of Motor and Process Skills (AMPS)* [33]. The AMPS is a standardised, observation-based assessment designed to evaluate the quality of a person’s ADL performance regarding ease, efficiency, safety, and independence. A trained and calibrated P-OT observes the quality of 16 motor and 20 process performance skills (ADL ability) while the person performs two familiar and relevant ADLs, and rates the person’s performance of each skill on a four-point ordinal scale. The ADL motor ability is a measure of how much physical effort, clumsiness, and/or fatigue the person demonstrates during ADL performance. The ADL process ability is the person’s overall efficiency regarding appropriate use of time, space, and objects throughout ADL performance. The ordinal scores are converted into two overall linear ability measures by Rasch-based computer-scoring software: one for ADL motor ability and one for ADL process ability expressed in logistically transformed probability units (logits). The two overall linear ability measures are adjusted for task challenge, skill item difficulty, and rater severity. ADL motor ability above 2.0 logits and ADL process ability above 1.0 logits indicate competent ADL performance, and 0.3 logits a clinical relevant change [33]. In the present study, the ADL motor ability is used as the primary outcome. The P-OT also assesses the five most effective and the five most ineffective ADL motor and process skills based on clinical reasoning [33]. The AMPS has been found valid and reliable in people with advanced cancer [34, 35] and responsive in people with other disorders [33].

### Secondary outcomes

#### Problems with everyday activities prioritised to be solved

Problems with everyday activities at home that the participants face and prioritise to have solved, will be assessed using the *Individually Prioritised Problems Assessment (IPPA)* [36]. The instrument has a structured interview format where the participant identifies up to seven everyday activity problems and rates the importance of and ease/difficulty with each of these problems on five-point ordinal scales from 1 to 5, where 1 = not important at all and 5 = most important; and 1 = no difficulty at all and 5 = too much difficulty to perform the activity at all. The importance scores and the difficulty scores of each activity are multiplied. The scores are then added up and divided by the number of activity problems, resulting in an average IPPA score between 1 and 25. Higher average IPPA scores indicate more difficulty with the prioritised everyday activities [36]. The IPPA has been found to be a useful, responsive, and valid instrument in older people who use assistive devices [37, 38].

#### Autonomy and participation in the dwelling

Autonomy and participation are assessed using three subscales of the Danish version (IPA-DK) of the *Impact on Participation and Autonomy Questionnaire (IPAQ)* [39, 40]. The IPA-DK is a questionnaire targeting adults with chronic functional limitations. It assesses person-perceived participation restrictions via 32 items organised into five subscales: 1. Autonomy indoors, 2. Family roles, 3. Autonomy outdoors, 4. Social life and relationships, and 5. Work and education. For this study, the subscales 1, 2, and 4 are used. Response options are given on five-point ordinal scales from 0 = very good to 4 = very poor. In addition, there are nine items which quantify the degree to which the respondents perceive these restrictions as problematic in their daily life. As these are used for clinical decision making, they are not included in this study. IPAQ is available in several language versions which have well-documented psychometric properties, and the Danish version has undergone a reliability test with satisfactory results [39].

#### Health-related quality of life

Health-related quality of life will be assessed by means of *The European Organization for Research Treatment of Cancer Quality of Life Questionnaire Core 30 (EORTC QLQ C-30)* [41], designed to assess the health-related quality of life of persons with cancer. The instrument consists of nine scales assessing: physical function, role function, emotional function, cognitive function, social functioning, global health status/quality of life, fatigue, nausea and vomiting, and pain, and six single-item scales: dyspnoea, insomnia, lack of appetite, constipation, diarrhoea, and financial difficulties. All the scales and single-item measures range in a score from 0 to 100. Higher score represents a higher (“better”) level of functioning or a higher (“worse”) level of symptoms [41]. The EORTC QLQ C-30 is a well-validated and reliable instrument within cancer research [42, 43]. Additionally, health-related quality of life will be measured by the use of the *EuroQol instrument (EQ-5D-5L)* [44]. The EQ-5D-5L is a generic instrument measuring health-related quality of life, and it is widely used in economic evaluations. The instrument consists of five dimensions: mobility, self-care, usual activities, pain/discomfort and anxiety/depression. The participant scores each dimension on a five-point ordinal scale ranging from no problems to severe/extreme problems. A Danish set of preference values was constructed based on interviews with 1332 Danish respondents [45]. The psychometric properties of the EQ-5D-5L including the Danish version have been extensively investigated and are considered as good [46]. The data from the EQ-5D-5L will be used for the economic evaluation alongside the RCT.

### Descriptive data

#### Everyday activity pattern

The everyday activities that the participants engage in during a day will be captured by the Time Geographical method using a semi-structured diary [47]. The participants will be required to record the activities they undertake during one day of their own choice. The diary encompasses domains regarding which activities the participants engage in during the day, at what time, for how long, whom they are with, where they are, and how they feel physically and mentally [47].

#### Joyful activities

As a way to better understand what activities are of importance to the participants they are asked which everyday activities they regard as especially joyful [48].

#### Experienced usefulness

Participant observations will be conducted during 10–20 intervention sessions depending on the number of interventions provided for the individual participants [49]. In addition, telephone interventions provided to this sub-sample will be electronically recorded. In particular, attention will be on the participants’ reactions to the intervention as it takes place.

Qualitative interviews will be conducted in conjunction with data collection at follow-up in the participants’ homes. The interviews that will explore the usefulness of the intervention will be based on an interview guide developed from a preliminary analysis of the previous participant observations to acquire the participants’ experiences of the intervention received and its usefulness.

### Cost data

#### Intervention costs

The intervention costs will be measured based on micro costing including occupational therapists’ and study participants’ time spent on the intervention; the costs for applications and assistive devices and home modifications; the occupational therapists’ time used for related administrative purposes and transportation.

#### Costs in the secondary health care sector

The costs of secondary health care will be determined by Diagnostic Related Group (DRG) tariffs extracted from the National Patient Registry (NPR). The data include information on hospital departments, dates of admission and discharge, and diagnosis [50].

#### Costs in the primary health care sector

Data on the use of primary health care including contacts to general practitioners, medical specialists, and physiotherapists will be extracted from The Danish National Health Service Register for Primary Care (NHSR) and valued using the activity-based fees that are used to reimburse these providers. The NHSR contains information about the activities of health professionals’ contacts with the tax-funded public health care system [50].

#### Prescriptive medication

Data on the use of prescriptive medication will be extracted from the Danish National Register of Prescriptive Medication. This database includes information on all redeemed prescriptive medication and the associated costs.

#### Study participants’ out-of-pocket costs

Out-of-pocket costs such as non-prescriptive medication, dietary supplements, informal care, aids, and short term sick leave are assessed using a modified version of the Dutch cost diary [51]. The patient out-of-pocket costs will solely be included in the sensitivity analysis.

#### Productivity costs

This will be calculated using data on the number of weeks of sick leave obtained from the Danish Register for Evaluation of Marginalization (DREAM), which is administered by the Danish Ministry of Employment. This database includes information on all public transfer payments for all Danish citizens registered on a weekly basis since 1991 [52]. The productivity costs per study participant will be calculated using the Human Capital method [53]. Productivity costs will solely be included in the sensitivity analysis.

## Sample size

Two hundred and seventy two participants will be enrolled. The sample size calculation was based on the mean ADL motor ability as identified in the cross-sectional study to be 1.04 logits with a standard deviation (SD) of 0.727 logits [18]. For a two-sample *t*-test of normal distribution with a two-sided significance level of 0.05 and a common SD of 0.727, a sample size of 184 participants (92 per group) would provide 80 % power to detect a between-group difference of 0.3 logits [33]. To achieve this number, the study needs a total of 272 participants (136 per group), expecting a dropout rate based on previous studies of 32 % at 12 weeks follow-up [14, 16, 24, 54].

A sub-sample of ten participants will be recruited among the entire sample using purposive sampling for participant observations during intervention sessions and for qualitative interviews [48]. Specific criteria for selection will be defined prior to actual sampling.

## Data collection

**Fig. 1:**
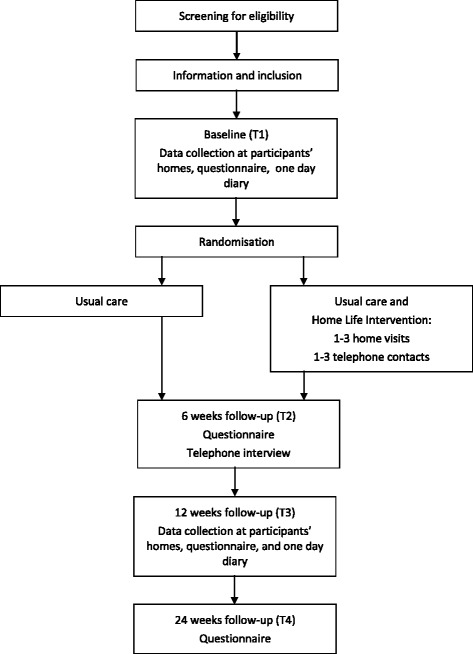
Randomisation and data collection

Data will be collected at baseline (T1) by means of a study specific questionnaire (demography, health, cost diary, the IPA-DK, EORTC QLQ C-30, and the EQ-5D-5 L) and the One Day Diary. The questionnaire and the One Day Diary are sent out before a home visit by the P-OTs, where the AMPS and IPPA are applied, and a question about joyful activities and one about how they manage their everyday life are asked. In addition, data concerning use of assistive devices using a study specific questionnaire are collected. The two ADLs that the participant will perform for the AMPS observation are selected on basis of the One Day Diary and the IPPA data. See Fig. 1 and Table 1. The quality of AMPS data will be monitored by Center for Innovative OT Solutions, USA, and only valid data will be included.

**Table 1:** Schedule for collection of outcomes and cost data

Outcome	Source	Data collection method	Baseline T1	Week 6 T2	Week 12 T3	Week 24 T4
Everyday activities						
Quality of ADL performance	AMPS	Observation	X		X	
Problems with everyday activities	IPPA	Structured interview	X	X	X	
Autonomy and participation	IPA-DK	Questionnaire	X	X	X	
Health-related Quality of Life						
Health-related quality of life	EORTC QLQ C-30	Questionnaire	X	X	X	
Economic evaluation						
Quality Adjusted Life Years (QALY)	EQ-5D-5L	Questionnaire	X	X	X	X
Intervention costs		Questionnaire		X		
Costs in the secondary health care sector	The National Patient Registry	Register	X	X	X	X
Costs in the primary healthcare sector	The Danish National Health Service Register for Primary Care	Register	X	X	X	X
Prescriptive medication	Danish Register of Prescriptive Medication	Register	X	X	X	X
Out-of-pocket costs		Cost diary	X	X	X	X
Productivity costs	The Danish Register for Evaluation of Marginalization	Register and questionnaire	X	X	X	X

*AMPS* The Assessment of Motor and Process Skills, *EORTC QLQ-C30* The European Organization for Research Treatment of Cancer Quality of Life Questionnaire Core 30, *EQ-5D-5L* The EuroQol 5-dimensions 5 levels, *IPA-DK* The Danish version of the Impact on Participation and Autonomy Questionnaire (IPAQ), *PPA*, The Individually Prioritised Problems Assessment

There will be three follow-up occasions where cost data from registers are also extracted, while demographic data are only collected at T1:

T2) Six weeks after T1: a postal questionnaire identical to the one sent out at T1 and a study specific questionnaire on the type of interventions both groups have received and/or completed. Besides, an IPPA telephone interview is accomplished.

T3) 12 weeks after T1: identical to T1 except that a question whether their everyday life has improved is also asked.

T4) 24 weeks after baseline: a postal questionnaire that includes the cost diary and the EQ-5D-5L.

Observation based data are collected in conjunction with the intervention, and qualitative interviews with a subsample are accomplished at T3.

## Randomisation

Study participants will be randomly assigned to either the intervention group (receiving the Cancer Home-Life Intervention as a supplement to usual care) or the control group (receiving usual care and not receiving the Cancer Home-Life Intervention). See Fig. 1.

Randomisation will be carried out after T1 by an administration office, which is independent of the trial. Participants will be randomly assigned in a preset block size to either the intervention or control group with a 1:1 allocation by a computer-generated randomisation schedule. The block size will be kept unknown to all investigators and P-OTs and will not be revealed until the study has ended. The randomisation is stratified by centre.

## Blinding

Allocation is concealed to the study investigators. The P-OTs will also be sought blinded for the group allocation even though they may discover this when collecting follow-up data, and the study participants are told not to reveal their group allocation to the P-OTs during the follow-up occasions. At T3 the P-OTs will be asked to guess the group allocation of the participants. This may indicate if the blinding has succeeded. Allocation status cannot be blinded for the participants.

## Analysis

### Data analyses of the clinical evaluation

Scores will be calculated following the instructions in each instrument manual and presented by descriptive statistics. The intervention group will be compared with the control group by means of a multiple linear regression analysis in continuous data (AMPS, EORTC QLQ C-30 and IPPA), given the assumptions are fulfilled. If the assumptions are not met, a relevant transformation or logistic regression will be used. IPA-DK data are ordinal and will be dichotomised before logistic regression analysis is applied. The analysis of all outcomes will be adjusted for the stratification variable, centre. If there is significant imbalance between the two groups we shall consider adjusting for baseline ADL motor ability, baseline ADL process ability, gender, age, primary cancer diagnosis, education, employment and the EORTC QLQ-C30 global health status/quality of life in a sensitivity analysis [55, 56]. The between-group differences in continuous and dichotomised data will be presented with 95 % confidence intervals. A complete case analysis excluding participants without post-randomisation data will be performed [57]. It will be supplemented with a sensitivity analysis applying multiple imputation used to estimate a plausible value for the missing data of study participants lost to follow-up of other reasons than death [58].

Analyses will be performed to identify groups in which the Cancer Home-Life Intervention is especially effective. A subgroup-treatment effect interaction by a multiple regression analysis will be performed with the following possible variables as effect modifiers: age, gender, primary cancer diagnosis and WHO Performance score [59, 60].

*P* values ≤0.05 will be considered statistically significant. Analyses will be performed using STATA.

The One Day Diary data from T1 and T3 will be coded thematically into the following structure: 1) The activity domain comprising seven categories: self-care; care for others; household; leisure; transportation; procurement and preparation of food; and work. 2) The geographical domain: location and movements. 3) The social context: social circle and interaction. 4) The experiential domain: physical state and state of mind. The analyses will be used to describe the study participants’ daily activities with regard to each domain including duration and frequency of activities as well as activity patterns for the individual over the time of a day [47].

Field notes from the participant observations will be transcribed into a coherent text. Interviews will be transcribed verbatim. Both data sets will be analysed by thematic analysis to unfold and understand how the participants reacted to and experienced the usefulness of the received interventions, and to explore how the intervention worked and what aspects had particular relevance for the participants [48]. Thereafter, data derived from participant observations and interviews will be analysed with a constant comparative method. The analysis involves comparing different types of data from the two methods in order to systematically trace out categories and relationships within the data [61].

### Data analysis for the economic evaluation

The economic evaluation will be conducted as a cost-effectiveness analysis. A health sector viewpoint will be taken to estimate the costs of all activities and resource use related to the study participants’ disease. T1 will be taken as the start of the time frame that will end at T4. All costs will be reported in 2015-Euros. By the use of register data to estimate costs, we expect a full follow-up of these data. The method of multiple imputation [58] will be used to handle possible lost to follow-up in the ADL motor ability or the EQ-5D-5L due to other reasons than death.

In the cost-effectiveness analysis the ADL motor ability will be used as the clinical parameter and QALY will be used as the measure of utility. In order to calculate QALY the EQ-5D-5L the recommended standard mapping procedure will be used based on the Danish preference weights [45]. The QALYs over 24 weeks will be calculated by interpolation of the area under the curve with four time points (T1, T2, T3, and T4). The resource use, costs, and clinical outcome will be presented as means with 95 % bootstrapped confidence intervals (10,000 replicates) [62]. The Incremental Cost-Effectiveness Ratio (ICER) will be calculated using the formula: ICER = (C_A_ – C_B_) / (E_A_ – E_B_), where C denotes costs and E denotes effects with A and B referring to comparators. The ICER summarises the results of each economic evaluation in a single parameter, defined as the ratio of additional costs per additional unit of effect [53]. The ICER is, however, undefined if the ratio or just one of the confidence limits is negative.

The result of the cost-effectiveness analysis will be summarised in cost-effectiveness acceptability curves (CEAC) [63]. A CEAC is a graphic representation of the uncertainty in cost differences and effect differences between the two groups [63]. To deal with the structural uncertainties, sensitivity analysis will be performed to test the influence of the chosen imputation strategy. Further sensitivity analysis will be performed to test the inclusion of productivity costs and patients’ out-of-pocket payments [53].

## Ethical considerations

All participants enrolled in the project will receive written and oral information about the project procedures and will have volunteered to participate, which will be verified by written consent. All eligible participants will be informed that they are free to withdraw from the study at any time without consequences for their future care. The trial will be conducted in accordance with the ethical principles outlined in the Declaration of Helsinki 2008 [64]. According to the Danish Regional Scientific Ethical Committee regulations the project is not notifiable, because no human biological material is included in the project (S-20122000-96). Permission to obtain and store data was originally given by the Danish Data Protection Agency (J.nr. 2012-41-1404), but controllership has later (March 27 2015) been transferred to the umbrella/joint notification of Southern Denmark to the Danish Data Protection Agency of University (FN 215-57-0008). The intervention group will be treated by authorised occupational therapists trained specifically for the present study. Since the control group will be offered the care that is usually given, and the outcome of the Cancer Home-Life Intervention is expected to have a positive impact on the everyday activities of people with advanced cancer, allocation to either the control group or the intervention group is ethically acceptable. No adverse effects of the planned intervention are expected. However, because the participants suffer from a life-threatening disease, the assessments may cause emotional reactions [65]. This will be handled in an ethically appropriate manner: all participants will receive a telephone number so that they can contact the research team if needed, and time is allocated for the P-OTs and the I-OTs to talk to the participants if required. All results will be handled confidentially, and only group results will be published.

The project is approved by the Danish Data Protection Agency (J.nr. 2012-41-1404). Data will be stored in locked filing cabinets or in password-protected computers at the University of Southern Denmark, archived in accordance with University guidelines and the Danish Data Protection Agency.

The study is registered in www.controlled-trials.com/ClinicalTrials.gov (NCT02356627).

## Discussion

The present study will contribute with knowledge about whether the Cancer Home-Life Intervention can support people with advanced cancer living at home in performing and participating in prioritised everyday activities, and whether the intervention contributes to their health-related quality of life. So far knowledge about improving and preserving everyday activities of people with advanced cancer is scarce. Given that these people live longer and are increasingly receiving medical treatment on an outpatient basis, they need to be able to manage or live an everyday life according to their own wishes; there is a definite need for such knowledge.

A strength of the study is that parts of it are based on the previous cross-sectional study serving as a kind of feasibility study as recommended by The Medical Research Council [66]. For instance we gained experience with ways of recruiting from hospitals, time use and procedures for data collection; we got knowledge about the demographics and clinical characteristics of the study population and which everyday activities they had problems with, and which they would like to have solved. Most importantly the cross-sectional study provided empirical information that could be used to calculate the sample size on basis of our primary outcome measure. The calculation was therefore based on the required number of participants at T3, taking expected drop-out into account in order to get sufficient power to detect possible effects. Another strength is that we involved a patient expert group consisting of representatives of people with advanced cancer to advise us about the contents and feasibility of the Home-Life Intervention and piloted the procedures and the intervention prior to trial start (*N* = 4).

One of the challenges encountered in the cross-sectional study was the mortality of the study population. For instance only ten of the 84 study participants included from OUH were alive one and a half year after completion of the cross-sectional study. The consequence may be that a substantial part of the study participants may not be alive at the 24 weeks’ follow-up (T4). Since data from T4 are solely used for the economic evaluation and mostly consist of register based data, it still is possible to get useful results.

The study is one of the first to investigate the effect of everyday activity interventions for people with advanced cancer in the home. In addition to data on the effectiveness of the Cancer Home-Life Intervention, the study will provide information about the cost-effectiveness of the intervention, its acceptability for the target group, and whether some subgroups might benefit more than others from the intervention. Hence the study will yield comprehensive knowledge that can be applied in palliative care in case of positive results; if so, the next step will be to investigate how to implement the Cancer Home-Life Intervention in municipality palliative care.

## Abbreviations

ADL: Activities of daily living
AMPS: The Assessment of Motor and Process Skills
AUH: Aarhus University Hospital
CEAC: Cost-effectiveness acceptability curve
DREAM: The Danish Register for Evaluation of Marginalization
DRG: Diagnosis related group
EORTC QLQ-C30: The European Organization for Research Treatment of Cancer Quality of Life Questionnaire Core 30
EQ-5D-5 L: The EuroQol 5-dimensions 5 levels
ICER: Incremental Cost-Effectiveness Ratio
I-OT: Intervention Occupational Therapist
IPA-DK: The Danish version of the Impact on Participation and Autonomy Questionnaire (IPAQ)
IPPA: The Individually Prioritised Problems Assessment
NPR: The National Patient Registry
NHSR: The Danish National Health Service Register for Primary Care
OUH: Odense University Hospital
P-OT: Project occupational therapist
QALY: Quality Adjusted Life Years
RCT: Randomised controlled trial
WHO: World Health Organization
